# Supplementary figures and images for: On the role of VP3-PI3P interaction in birnavirus endosomal membrane targeting
Source: eLife. 2025 Mar 6;13:RP97261. doi: 10.7554/eLife.97261 (PMC11884790; doi:10.7554/eLife.97261)

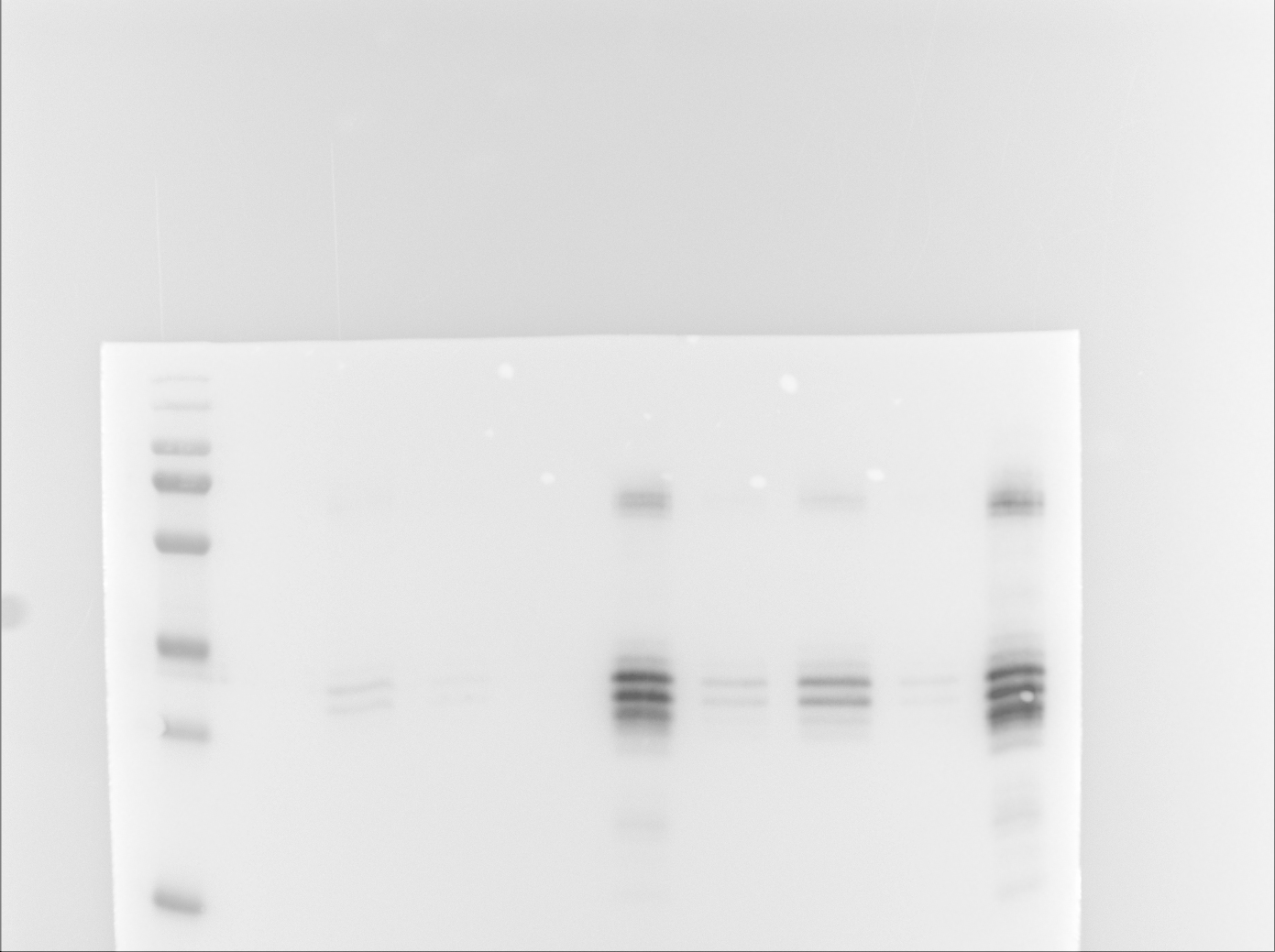

Supplement: Figure 1—source data 2. [file elife-97261-fig1-data2.zip › Figure 1 - Source Data 2/Figure 1B, His-VP3 FL.tif]

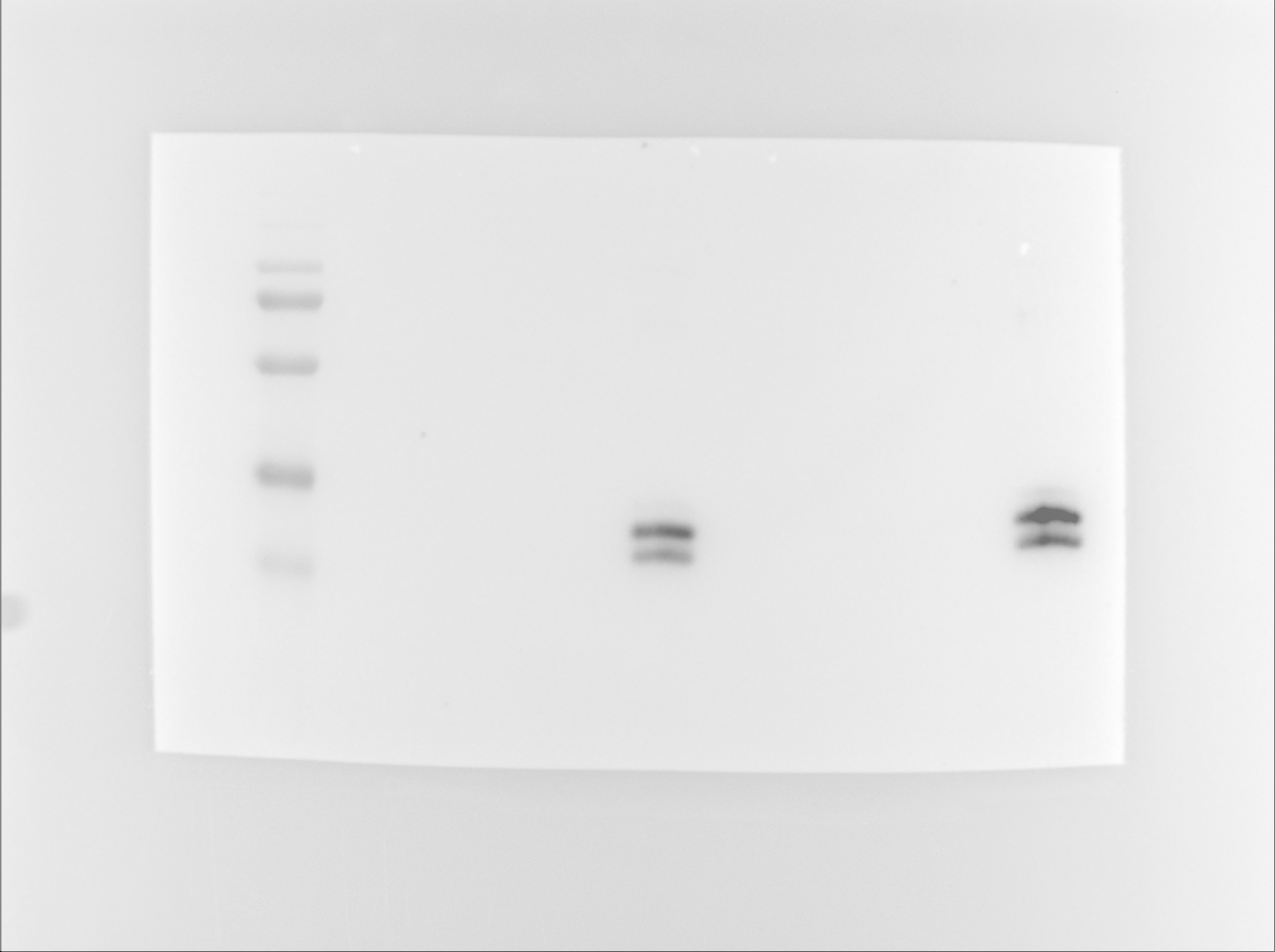

Supplement: Figure 1—source data 2. [file elife-97261-fig1-data2.zip › Figure 1 - Source Data 2/Figure 1G, His-VP3DeltaCT.tif]

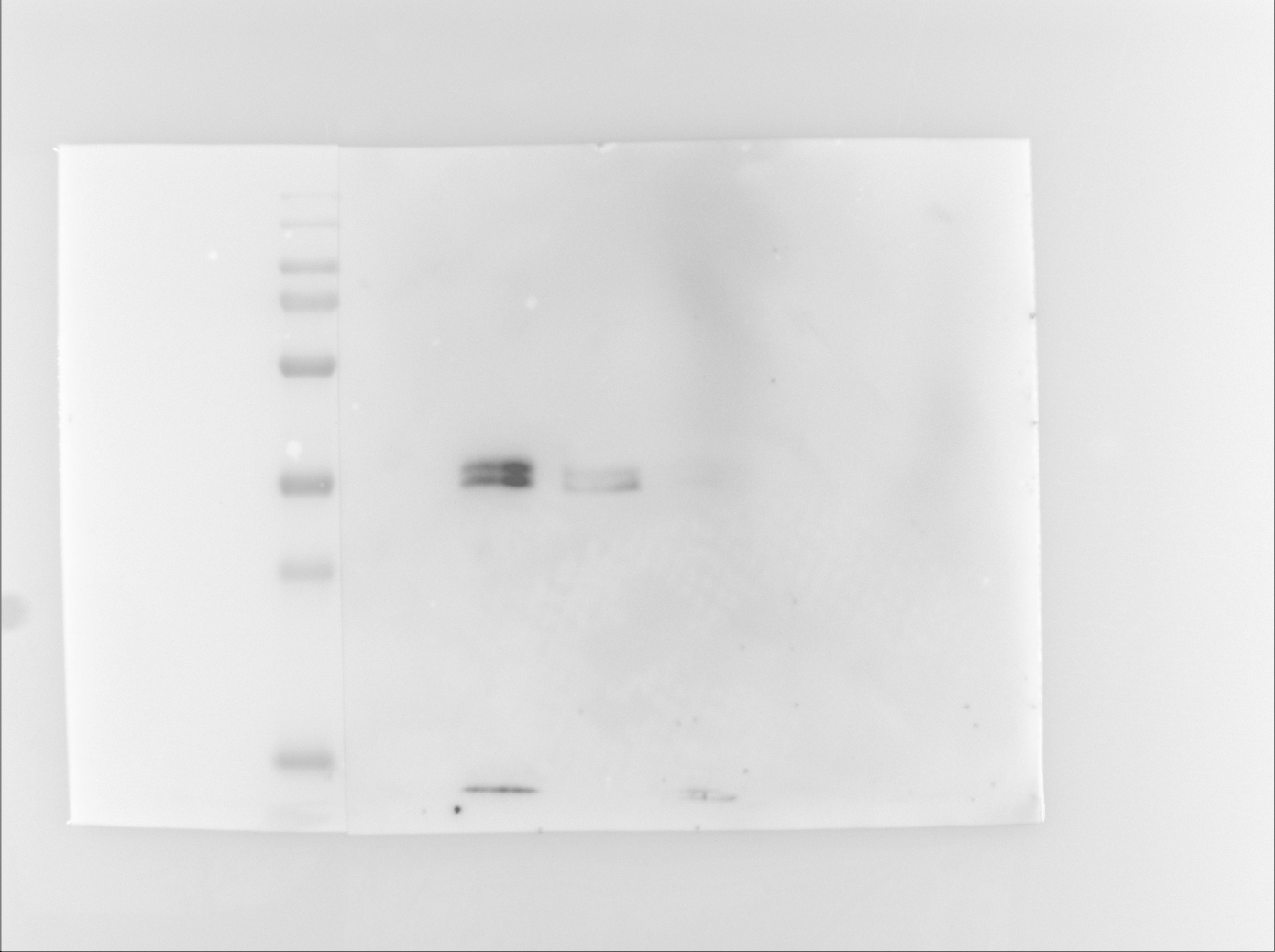

Supplement: Figure 1—source data 2. [file elife-97261-fig1-data2.zip › Figure 1 - Source Data 2/Figure 1A, His-2xFYVE.tif]

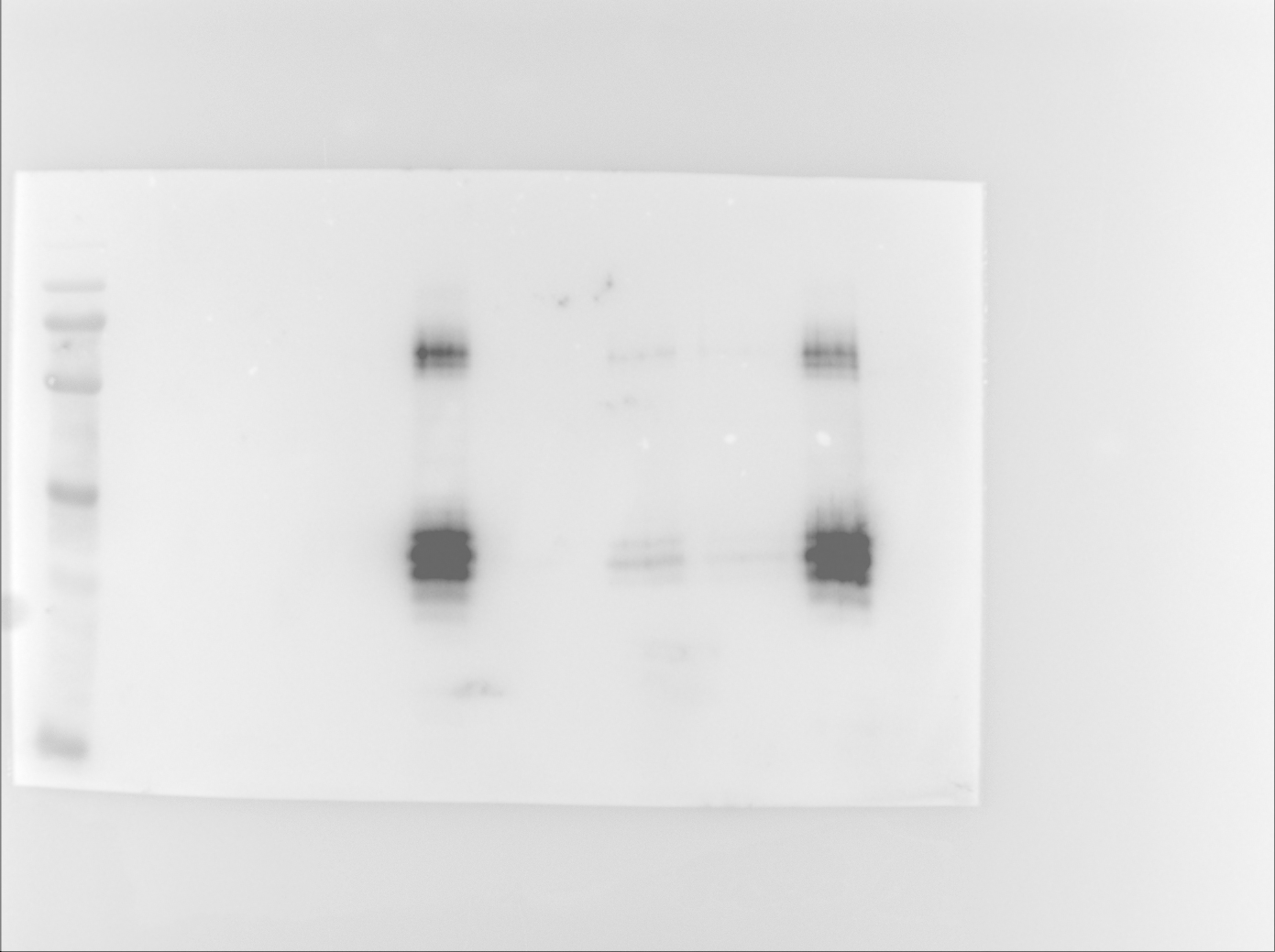

Supplement: Figure 1—source data 2. [file elife-97261-fig1-data2.zip › Figure 1 - Source Data 2/Figure 1G, His-VP3 FL.jpg]

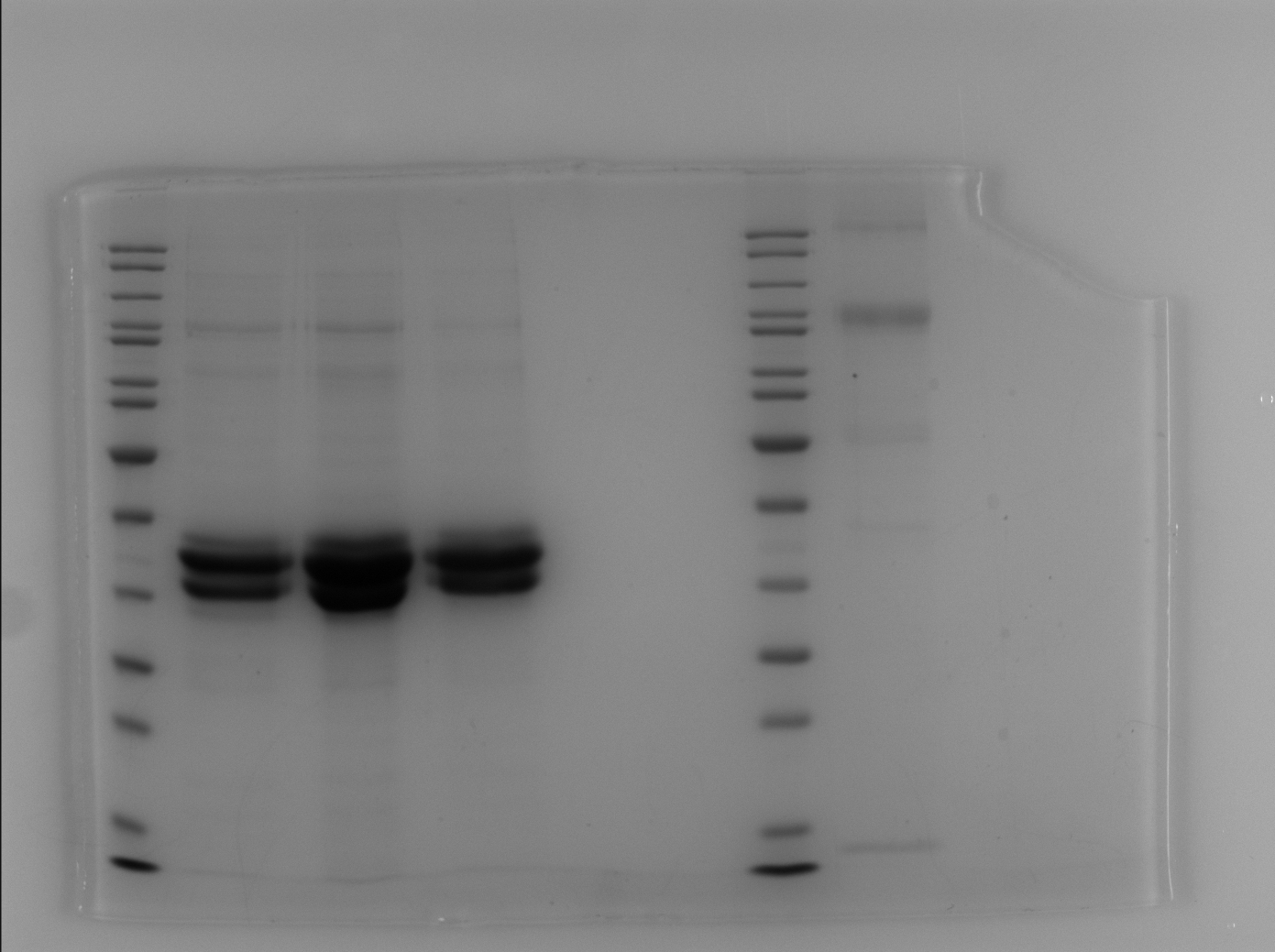

Supplement: Figure 1—figure supplement 1—source data 2. [file elife-97261-fig1-figsupp1-data2.zip › Figure S1 - Source Data 2/Coomassie Gel_His-VP3 FL.jpg]

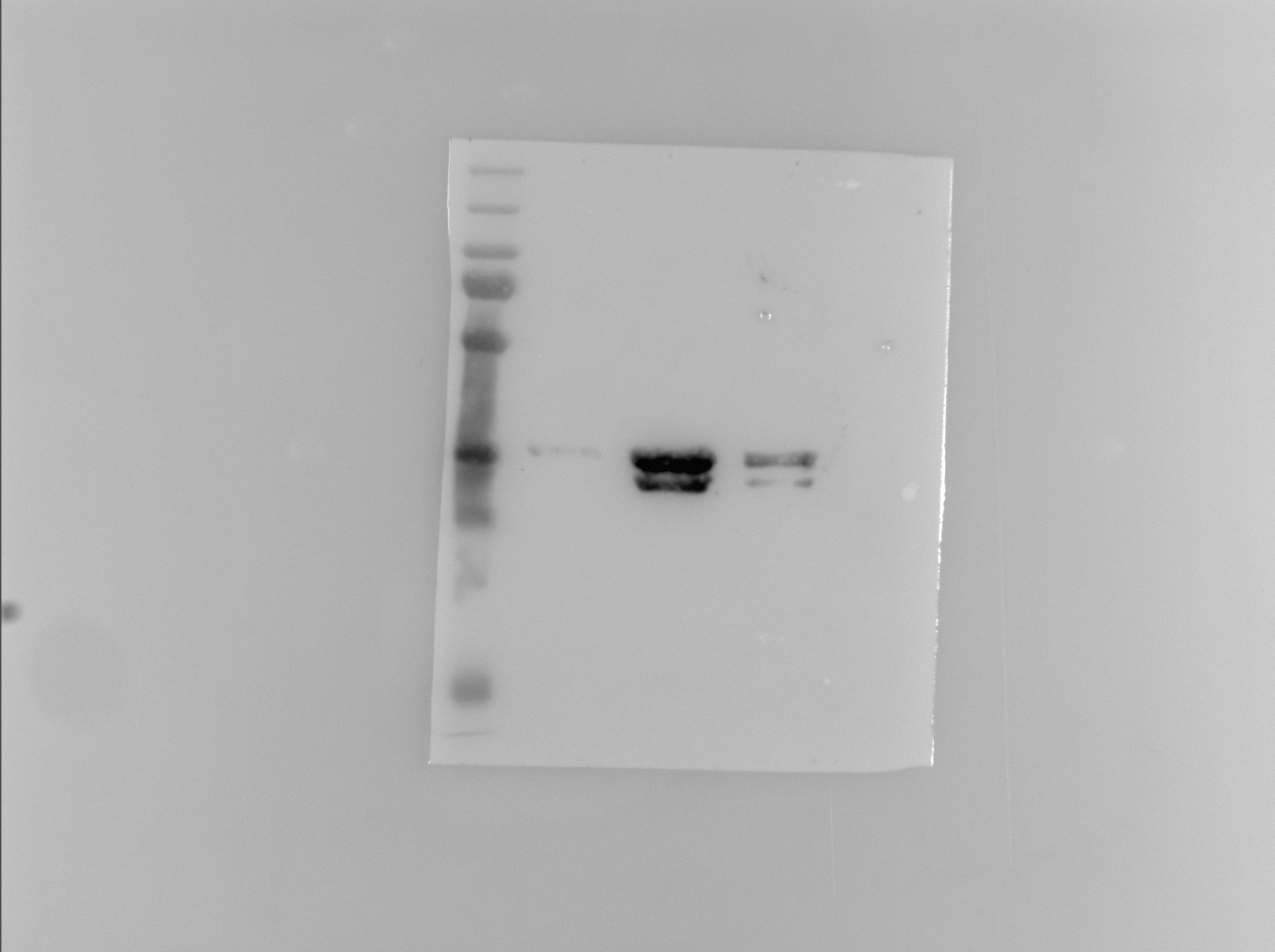

Supplement: Figure 1—figure supplement 1—source data 2. [file elife-97261-fig1-figsupp1-data2.zip › Figure S1 - Source Data 2/Western blot anti-His.jpg]

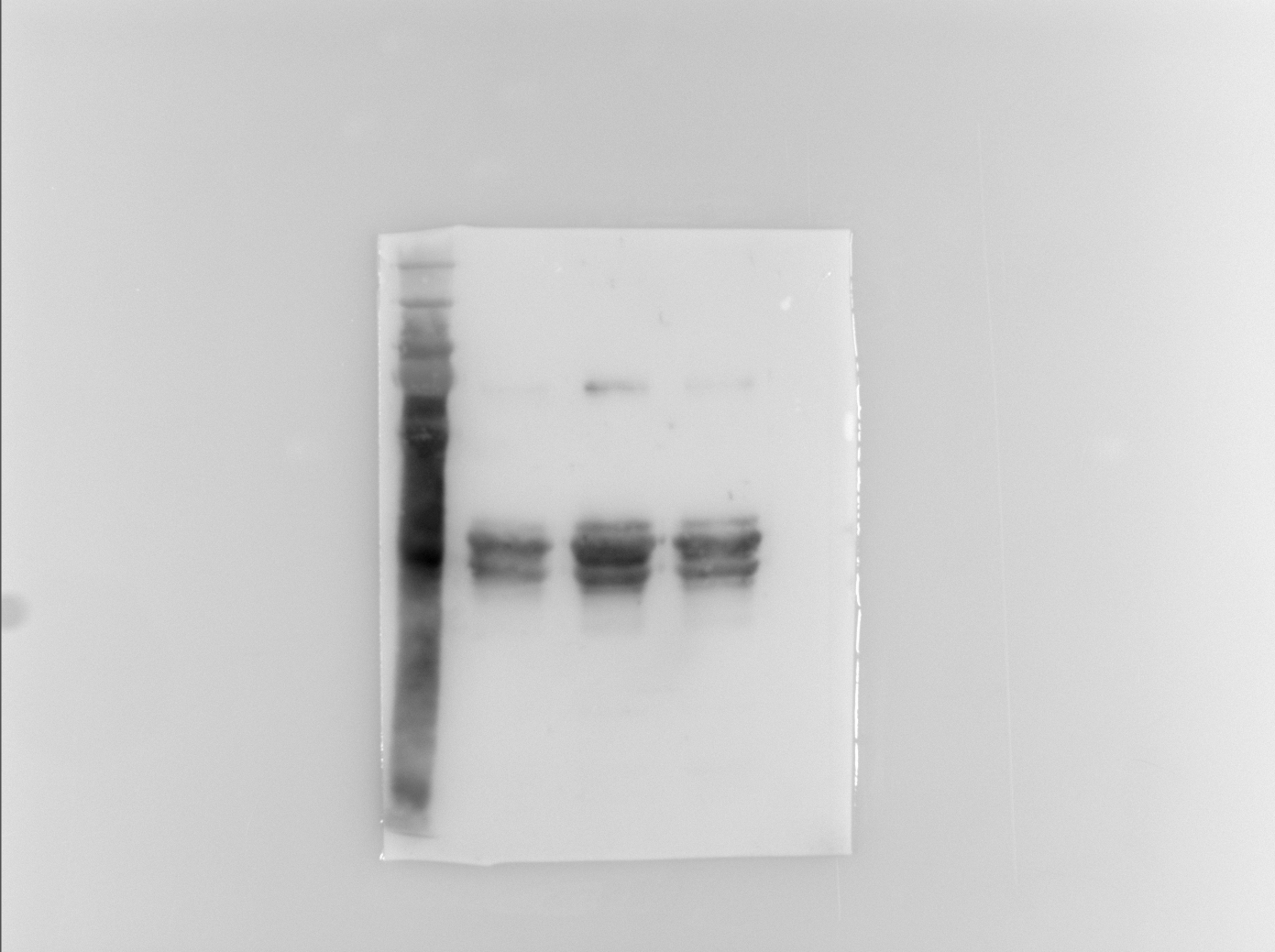

Supplement: Figure 1—figure supplement 1—source data 2. [file elife-97261-fig1-figsupp1-data2.zip › Figure S1 - Source Data 2/Western blot anti-VP3.jpg]

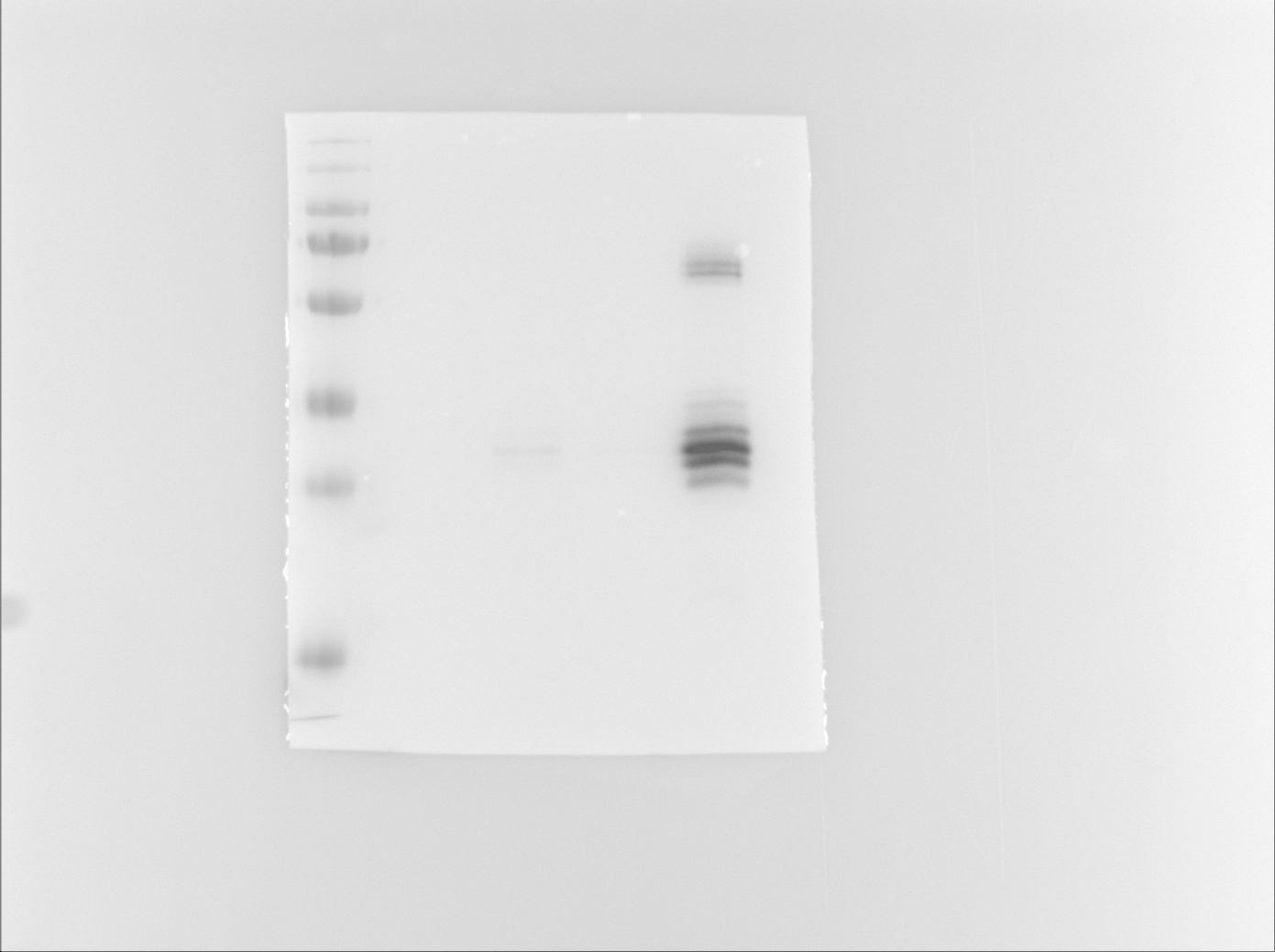

Supplement: Figure 1—figure supplement 2—source data 2. [file elife-97261-fig1-figsupp2-data2.zip › Figure S2 - Source Data 2/PI.tif]

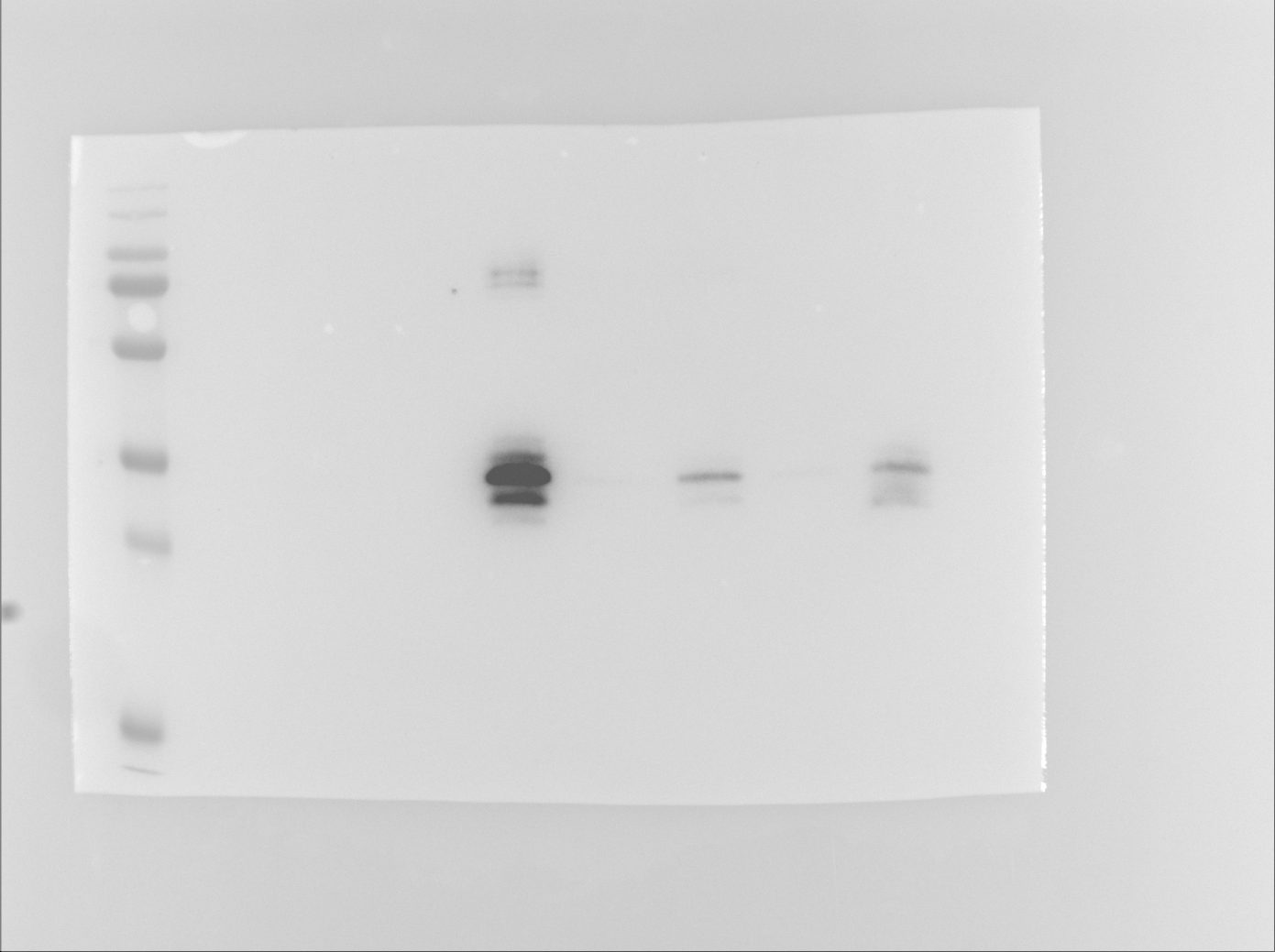

Supplement: Figure 1—figure supplement 2—source data 2. [file elife-97261-fig1-figsupp2-data2.zip › Figure S2 - Source Data 2/PA + PI3P.tif]

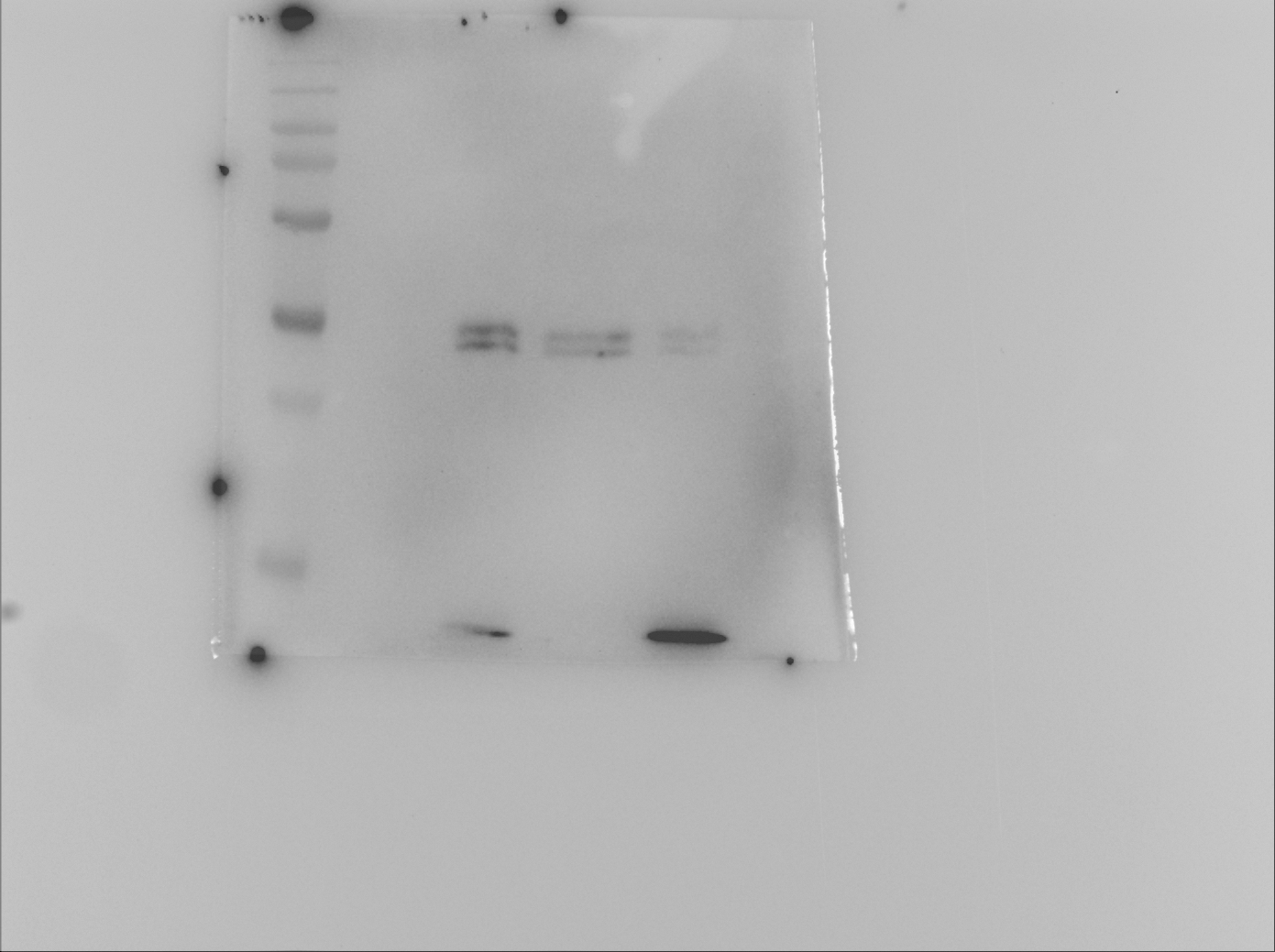

Supplement: Figure 4—source data 2. [file elife-97261-fig4-data2.zip › Figure 4 - Source Data 2/Figure 4A, His-2xFYVE.jpg]

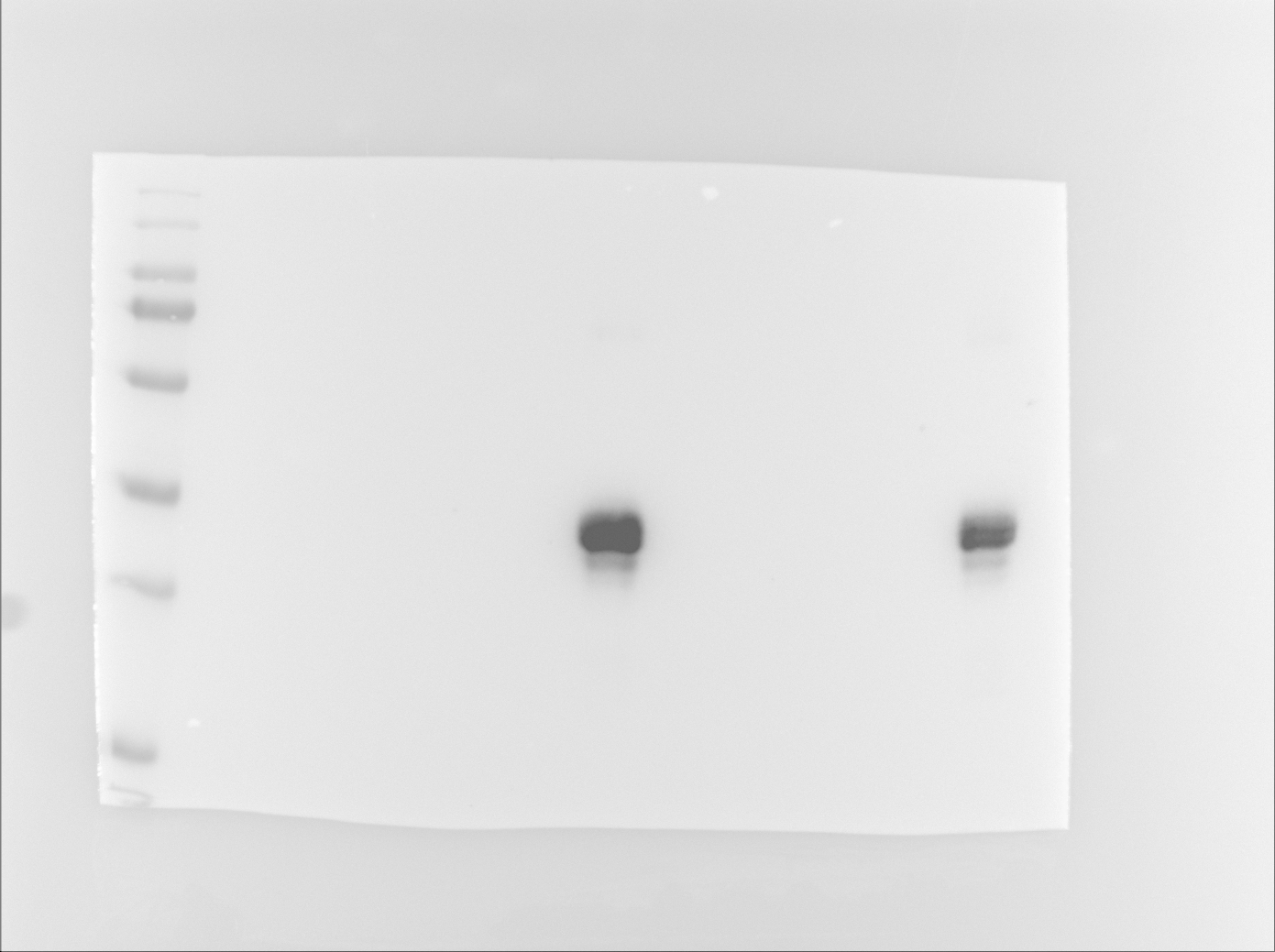

Supplement: Figure 4—source data 2. [file elife-97261-fig4-data2.zip › Figure 4 - Source Data 2/Figure 4A, His-VP3 FL R200D.jpg]

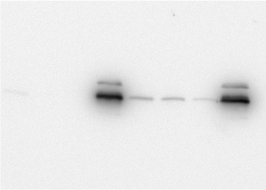

Supplement: Figure 5—figure supplement 1—source data 2. [file elife-97261-fig5-figsupp1-data2.zip › Figure S5 - Source Data 2/His-VP3 DeltaNt.png]

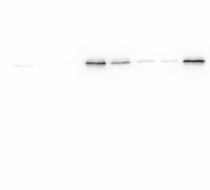

Supplement: Figure 5—figure supplement 1—source data 2. [file elife-97261-fig5-figsupp1-data2.zip › Figure S5 - Source Data 2/His-VP3 FL.png]
