# Supplementary material for: On the role of VP3-PI3P interaction in birnavirus endosomal membrane targeting
Source: eLife. 2025 Mar 6;13:RP97261. doi: 10.7554/eLife.97261 (PMC11884790; doi:10.7554/eLife.97261)
Supplement: Figure 1—figure supplement 1—source data 1. [file elife-97261-fig1-figsupp1-data1.pdf]

Figure 1—figure supplement 1- Source Data 1. Original Coomasie R. Blue-stained polyacrylamide gel and Western blot membranes corresponding to Figure S1, panel B and C.

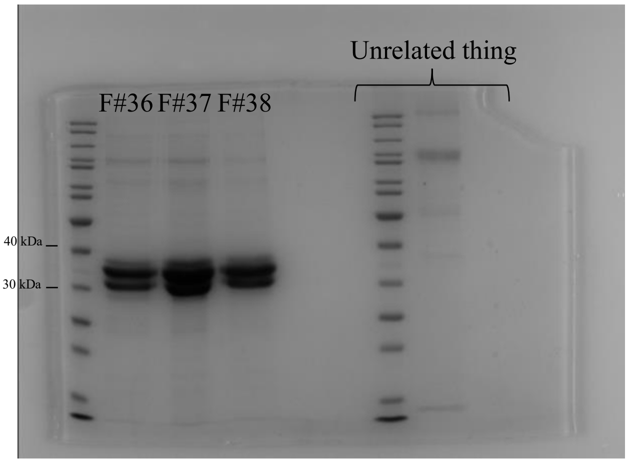

Panel B, Coomassie R. blue-stained polyacrylamide gel showing fractions (F) #36, #37 and #38 from the size exclusion chromatography of **His-VP3 FL** protein. The PageRuler Unstained Protein Ladder from Thermo Fisher Scientifc (Product #26614) was used.

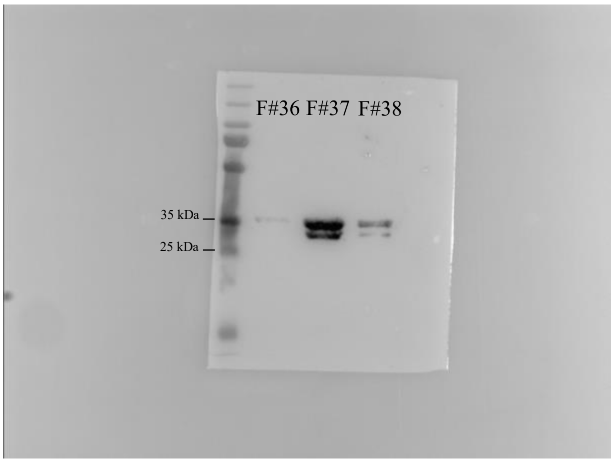

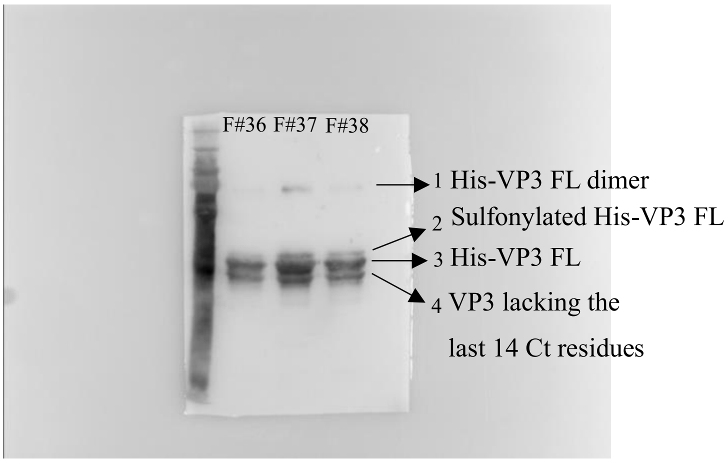

Panel C, Western blot images (left: anti-His; right: anti-VP3) from gels identical to that shown in (B). Numbers 1 to 4 indicate the identified proteins by mass spectrometry. In these cases, the Page Ruler Plus Prestained Protein Ladder from Thermo Fisher Scientifc (Product #26619) was used.
