# Supplementary material for: On the role of VP3-PI3P interaction in birnavirus endosomal membrane targeting
Source: eLife. 2025 Mar 6;13:RP97261. doi: 10.7554/eLife.97261 (PMC11884790; doi:10.7554/eLife.97261)
Supplement: Figure 1—figure supplement 2—source data 1. [file elife-97261-fig1-figsupp2-data1.pdf]

Figure 1—figure supplement 2 - Source Data 1. Original Western blot membranes corresponding to Figure Figure 1—figure supplement 2. In all the cases, the Page Ruler Plus Prestained Protein Ladder from Thermo Fisher Scientifc (Product #26619) was used.

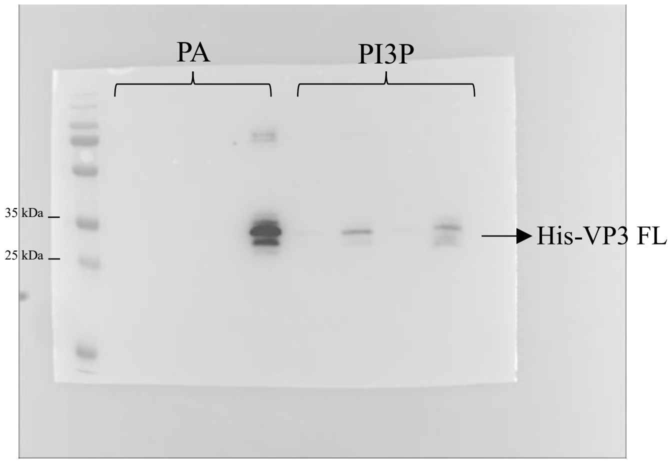

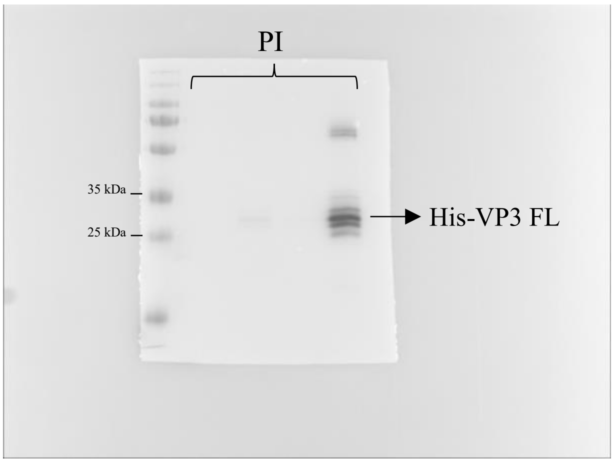

The membrane on the left contained the fractions from the flotation assay, where liposomes had been prepared with PA or PI3P and run together on the same gel. The membrane on the right contained the fractions from the flotation assay where liposomes had been prepared with PI only.
