# Supplementary material for: On the role of VP3-PI3P interaction in birnavirus endosomal membrane targeting
Source: eLife. 2025 Mar 6;13:RP97261. doi: 10.7554/eLife.97261 (PMC11884790; doi:10.7554/eLife.97261)
Supplement: Figure 4—source data 1. [file elife-97261-fig4-data1.pdf]

Figure 4 - Source Data 1. Original membranes corresponding to Figure 4, panel A. In both cases, the Page Ruler Plus Prestained Protein Ladder from Thermo Fisher Scientifc (Product #26619) was used.

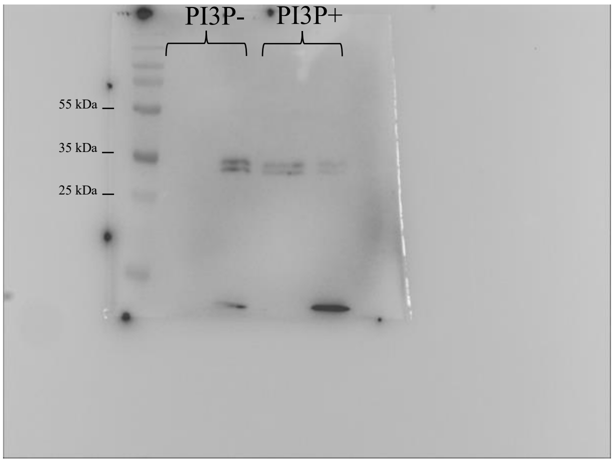

Panel A, **His-2xFYVE**

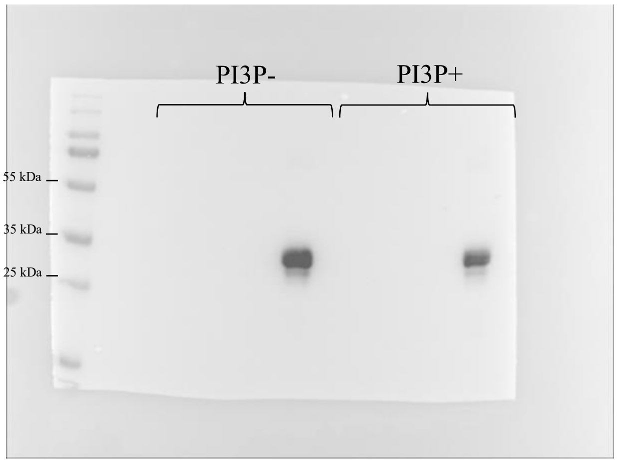

Panel A, **His-VP3 FL R200D**
