# Supplementary material for: On the role of VP3-PI3P interaction in birnavirus endosomal membrane targeting
Source: eLife. 2025 Mar 6;13:RP97261. doi: 10.7554/eLife.97261 (PMC11884790; doi:10.7554/eLife.97261)
Supplement: Figure 5—figure supplement 1—source data 1. [file elife-97261-fig5-figsupp1-data1.pdf]

Figure 5—figure supplement 1 - Source Data 1. Original Western blot membranes corresponding to Figure 5—figure supplement 1, panel A. We used the Page Ruler Plus Prestained Protein Ladder from Thermo Fisher Scientific (Product #26619) in the membranes. The membranes do not include the protein ladder, due to having observed that, sometimes, the anti-VP3 sera cross-reacted with certain bands from the ladder. This explains why in Figure 5—figure supplement 1, the ladder is shown separately.

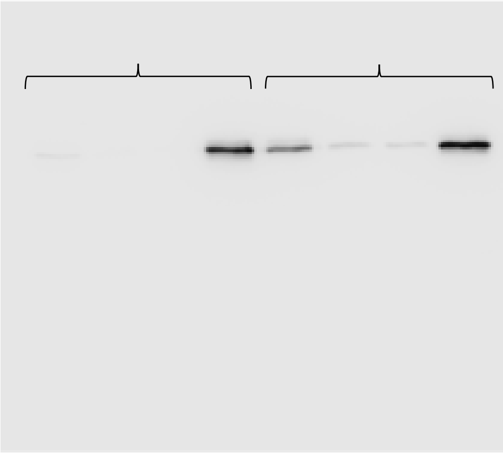

PI3P- PI3P+

Panel A, left membrane, **His-VP3 FL**

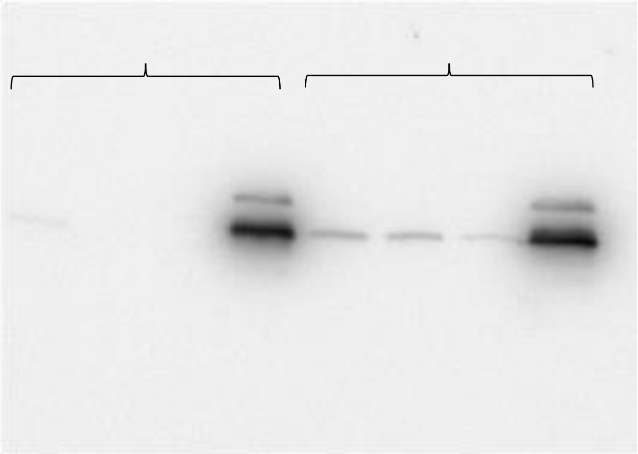

PI3P- PI3P+

Panel A, right membrane, **His-VP3 ΔNt**
