# Supplementary material for: On the role of VP3-PI3P interaction in birnavirus endosomal membrane targeting
Source: eLife. 2025 Mar 6;13:RP97261. doi: 10.7554/eLife.97261 (PMC11884790; doi:10.7554/eLife.97261)
Supplement: Supplementary file 1. — All the primers used for this work. * For the first four-point mutants, the nucleotides that allow to introduce amino acid changes in the VP3 protein are indicated in italic bold letters. The underlined nucleotides indicate restriction sites. [file elife-97261-supp1.docx]

| **Construction** | **Name of primer pairs** | **Sequences*** | **Position, mutation introduced or fragment** |
| --- | --- | --- | --- |
| pcDNA VP3 FL K157D | VP3.157 | 5’ ATGCAGAG***G***A***C***AGCCGGTTGGCATC 3’ (sense)  5’ AACCGGCT***G***T***C***CTCTGCATGCAC 3’ (antisense) | 157, K to D |
| pcDNA VP3 FL R159D | VP3.159 | 5’ GCATGCAGAGAAGAGC***GAC***TTGGCATCAG 3’ (sense)  5’ ***GTC***GCTCTTCTCTGCATGCACGTAGTCTAG 3’ (antisense) | 159, R to D |
| pcDNA VP3 FL H198D | VP3.198 | 5’ GTCTATGAAATCAAC***G***ATGGACGTGGC 3’ (sense)  5’ ***C***GTTGATTTCATAGACTTTGGCAACTTC 3’ (antisense) | 198, H to D |
| pcDNA VP3 FL R200D | VP3.200 | 5’ GAAATCAACCATGGA***GA***TGGCCCAAAC 3’ (sense)  5’ ***TC***TCCATGGTTGATTTCATAGACTTTGG 3’ (antisense) | 200, R to D |
| pFastBacHTb-his-VP3 FL | VP3.Fw  VP3.Rv | 5’ GGATCCGCTGCATCAGAGTTCAAAGAGAC 3’  5’ GAATTCTCACTCAAGGTCCTCATCAGAG 3’ | VP3 FL |
| pFastBacHTb-his-VP3 ∆223-257 | VP3.Fw  VP3.83-222.Rv | 5’ GGATCCGCTGCATCAGAGTTCAAAGAGAC 3’  5’ GAATTCTCAATTGCGATGCTTCATCTC 3 3’ | VP3 ∆223-257 |
| TrxA.His.Ts.2xFYVE | 2xFYVE | 5’ ACCGACGACGACGACAAGGAAAGTGATGCCATGTT 3’ (sense)  5’ GTGGTGGTGGTGCTCGAGGCCCGCGGTACCGTCGA 3’ (antisense) | 2xFYVE |
| TrxA.His.Ts.VP3FL | VP3.FL.Fw  VP3.FL.Rv | 5’ CTGGTGCCACGCGGTTCTGCATCAGAGTTCAAAGA 3’  5’ GCCCGCGGTACCGTCGACTTACTCAAGGTCCTCAT 3’ | VP3 FL |
| TrxA.His.Ts.VP3∆223-257 | VP3.FL.Fw  VP3.∆223257.Rv | 5’ CTGGTGCCACGCGGTTCTGCATCAGAGTTCAAAGA 3’  5’ GCCCGCGGTACCGTCGACTCAATTGCGATGCTTCA3’ | VP3 ∆223-257 |

Supplementary file 1. Primers
